# Radially varying viscosity and entropy generation effect on the Newtonian nanofluid flow between two co-axial tubes with peristalsis

**DOI:** 10.1038/s41598-023-37674-0

**Published:** 2023-07-07

**Authors:** H. A. Sayed, M. Y. Abouzeid

**Affiliations:** 1 Department of Mathematics, Faculty of Science, Zagazig University, Zagazig, Egypt; 2 Department of Mathematics, Faculty of Education, Ain Shams University, Heliopolis, Cairo Egypt

**Keywords:** Fluid dynamics, Mechanical engineering

## Abstract

To examine the peristaltic motion of a Newtonian fluid through an axisymmetric tube, many writers assume that viscosity is either a constant or a radius exponential function in Stokes’ equations. In this study, viscosity is predicated on both the radius and the axial coordinate. The peristaltic transport of a Newtonian nanofluid with radially varying viscosity and entropy generation has been studied. Under the long-wavelength assumption, fluid flows through a porous media between co-axial tubes, with heat transfer. The inner tube is uniform, while the outer tube is flexible and has a sinusoidal wave travelling down its wall. The momentum equation is solved exactly, and the energy and nanoparticle concentration equations are solved using the homotopy perturbation technique. Furthermore, entropy generation is obtained. The numerical results for the behaviours of velocity, temperature, and nanoparticle concentration, as well as the Nusselt number and Sherwood number with physical problem parameters, are obtained and graphically depicted. It is discovered that as the values of the viscosity parameter and the Prandtl number rise, so does the value of the axial velocity. Temperature values decrease as the wave amplitude and radiation parameter increase. Furthermore, at high values of the dependent viscosity parameter, the fluid nanoparticle gains more active energy and can move more freely, which is the main idea behind crude oil refinement. This physical modelling is essential for some physiological flows, such as the flow of stomach juice during the insertion of an endoscope.

## Introduction

Newtonian or non-Newtonian fluids are used by researchers, modellers, and physiologists at the early age as they do in many technical and medical sectors. By examining the behaviour of non-Newtonian distributions, they can apply engineering for oil reservoirs, material processing, and food production. A single relationship cannot be used to categorise all Newtonian liquids because to their disparate characteristics. Researchers have recently concentrated their attention on understanding how Newtonian or non-Newtonian fluids are utilised in the presence of nanoparticles. Implementations in the biomedical field, rheumatoid arthritis, digestive system, and oil refinement are a few examples^1–4^. The peristaltic transport of a Carreau fluid in a compliant rectangular duct was investigated by Riaz et al.^5^. Akram et al.^6^ demonstrate the effects of MHD hybrids on the thermal convection of Prandtl nanofluid flow. Other researchers investigate and discuss Newtonian and non-Newtonian nanofluid applications^7–19^.

Due to the importance of calculation in most phases, petrochemical chemicals and ink are two examples of fluids with variable viscosity. We agreed that fluid characteristics can alter in a suggestive way because of temperature variations. Investigational facts demonstrate, as an example, that the viscosity of water as represented in Table 1. The issue of peristaltic flow of a fluid with varying viscosity via a tube was examined by Eldabe et al.^20^. They also investigated the tube's centerline trapping phenomenon. By Nadeem et al.^21^, the effect of heat transfer in peristalsis with a viscosity of non-constant temperature is discussed. The peristaltic flow of non-constant viscosity in the presence of a chemical reaction was researched by Asghar et al.^22^. Eldabe et al.^23^ explore how a chemical reaction, nonconstant viscosity, and Ohmic dissipation affect the peristaltic motion of a pseudoplastic nanofluid.

**Table 1:** Values of water dynamical viscosity with temperature.

Temp. (°C)	2	3	4
Dyn. viscosity (mm^2^ g/ cm^3^ s)	1.6735	1.6190	1.5673

Nanoparticles and a carrier liquid are combined to form nanofluid. In addition, the fluid with nanoparticles has numerous engineering and technological applications, such as vehicle thermal management, vehicle cooling, heat exchangers, nuclear reactors, electronic device cooling, etc. In addition, the base fluid is typically a conductive fluid like oil, water, or ethylene glycol, and the nanoparticles are typically comprised of metals or non-metals. Thermal conductivity is higher for solid metals than for primary liquids. Suspended nanoparticles can thereby enhance thermal conductivity and heat transfer efficiency. Choi^24^ is credited with introducing the idea of nanofluids. Using a nanoparticle solution above a stretchable shape, Shafiq et al.^25^ investigated the issue of chemically reacting bioconvective of second-grade liquid. There are more studies^26–35^ that support further investigation in this area.

As a result of the investigations indicated above, the primary goal of this study is to present a new generalisation model for entropy generation and the impacts of changing viscosity parameters on the MHD peristaltic flow of biofluids. The peristaltic flow of Newtonian fluid is thought to be modelled by the blood flow through arterial catheterization. We made the long-wavelength and low-Reynolds number assumptions. Analytically approximating solutions to the momentum, energy, and nanoparticle concentration equations have been found using the homotopy analysis technique. Findings are graphically displayed and explained for various flow parameter problems. The gastric juice flow in the small intestine when an endoscope is placed is one physiological flow where this physical modelling is crucial.

## Problem formulation

Between two coaxial tubes and a porous media, we investigated the flow of Newtonian fluid. The transverse magnetic field B_0_ in the fluid is meant to be constant. While the outer tube's wall is being waved by a sinusoidal wave, the inner tube is rigid and homogenous. We employ the cylindrical coordinate system (*r*, *θ*, *z*). The inner and outer tube equations are as follows:

1 $$r_{{1}} = a,\;r_{2} = H = d + b\sin \frac{2\pi }{\lambda }(z - ct).$$

The equations that govern the flow are the balance of mass

2 $$\nabla \cdot \underline {V} = \, 0,$$

the equation of momentum

3 $$\rho (\underline{V} \cdot \nabla \underline{V} ) = - \nabla P + \nabla \cdot \underline{\tau } - \frac{{\mu_{B} }}{{k_{p} }}\underline{V} + \underline{J} \times \underline{B} ,$$

the equation of energy

4 $$\rho c_{p} (\underline{V} \cdot \nabla T) = k\nabla^{2} T - \nabla .\underline{q}_{r} + D_{T} \,\left( {\nabla T} \right)^{2} + D_{B} \,\left( {\nabla T} \right)\left( {\nabla C} \right),$$

the equation of concentration

5 $$(\underline{V} \cdot \nabla C) = D_{B} \,\nabla^{2} C + \frac{{D_{T} }}{{T_{2} }}\,\nabla^{2} T,$$

Maxwell^’^s equations

6 $$\nabla \times \underline{B} = \mu_{e} \underline{J} ,\,\nabla \cdot \underline{B} = 0,\;\nabla \times \underline{E} = 0,\;\nabla \cdot \underline{J} = 0$$

and Ohm’s law

7 $$\underline{J} = \sigma \,\left( {\underline{E} + \underline{V} \times \underline{B} } \right),$$

The governing equations for an incompressible flow in the fixed wave are given as^36–38^

8 $$\frac{\partial u}{{\partial r}} + \frac{u}{r} + \frac{\partial w}{{\partial z}} = 0,$$

9 $$\begin{aligned} \rho \left( {u\frac{\partial u}{{\partial r}} + w\frac{\partial u}{{\partial z}}} \right) & = - \frac{\partial P}{{\partial r}} + \frac{\partial }{\partial r}\left( {2\mu (r,z)\frac{\partial u}{{\partial r}}} \right) + \frac{\partial }{\partial z}\left( {\mu (r,z)\left( {\frac{\partial u}{{\partial z}} + \frac{\partial w}{{\partial r}}} \right)} \right) \\ & \quad - \frac{2\mu (r,z)}{r}\left( {\frac{\partial u}{{\partial r}} - \frac{u}{r}} \right) - \left( {\sigma B_{0}^{2} + \frac{\mu (r,z)}{K}} \right)u, \\ \end{aligned}$$

10 $$\begin{aligned} \rho \left( {u\frac{\partial w}{{\partial r}} + w\frac{\partial w}{{\partial z}}} \right) & = - \frac{\partial P}{{\partial z}} + \frac{\partial }{\partial z}\left( {2\mu (r,z)\frac{\partial w}{{\partial z}}} \right) + \frac{1}{r}\frac{\partial }{\partial r}\left( {r\mu (r,\,z)\left( {\frac{\partial u}{{\partial z}} + \frac{\partial w}{{\partial r}}} \right)} \right) \\ & \quad - \left( {\sigma B_{0}^{2} + \frac{\mu (r,z)}{K}} \right)w, \\ \end{aligned}$$

11 $$\begin{aligned} \left( {u\frac{\partial T}{{\partial r}} + w\frac{\partial T}{{\partial z}}} \right) & = \frac{k}{{\rho c_{p} }}\left( {\frac{{\partial^{2} T}}{{\partial r^{2} }} + \frac{1}{r}\frac{\partial T}{{\partial r}} + \frac{{\partial^{2} T}}{{\partial z^{2} }}} \right) - \frac{1}{{\rho c_{p} r}}\frac{{\partial (rq_{r} )}}{\partial r} + \frac{{\sigma B_{0}^{2} }}{{\rho c_{p} }}\left( {u^{2} + w^{2} } \right) + \\ & \quad + D_{T} \left( {\nabla T} \right)^{2} + D_{B} \left( {\nabla T} \right)\left( {\nabla C} \right) + Q_{0} \left( {T - T_{2} } \right), \\ \end{aligned}$$

12 $$u\frac{\partial C}{{\partial r}} + w\frac{\partial C}{{\partial z}} = D_{B} \left( {\frac{{\partial^{2} C}}{{\partial r^{2} }} + \frac{1}{r}\frac{\partial C}{{\partial r}} + \frac{{\partial^{2} C}}{{\partial z^{2} }}} \right) + \frac{{D_{T} }}{{T_{2} }}\left( {\frac{{\partial^{2} T}}{{\partial r^{2} }} + \frac{1}{r}\frac{\partial T}{{\partial r}} + \frac{{\partial^{2} T}}{{\partial z^{2} }}} \right).$$

The boundary conditions are given by:

13 $$u = 0,\,w^{\prime } = 0,\,T = T_{1} ,\,C = C_{1} \;{\text{at}}\;r = r_{1} .$$

14 $$u = - \frac{\partial H}{{\partial z}},\,w = - c,\,T = T_{2} ,\,C = C_{2} \;{\text{at}}\;r = r_{2} ,$$

By using the Rosseland approximation^39,40^, the radiative heat flux is given by:

15 $$q_{r} = \frac{{ - 4\sigma^{ * } }}{{3k_{R} }}\frac{{\partial T^{4} }}{\partial r}.$$

The temperature differences within the flow are small, such that *T*^4^ may be expressed as a linear function of temperature. This is accomplished by expanding *T*^4^ in a Taylor series about *T*_2_ and neglecting higher-order terms, one gets:

16 $$T^{4} \approx 4T_{2}^{3} T - 3T_{2}^{4} .$$

The appropriate non-dimensional variables for the flow are defined as

17 $$\begin{gathered} r^{ * } = \frac{r}{d},\;z^{ * } = \frac{z}{\lambda },\;u^{ * } = \frac{\lambda }{cd}u,\;w^{ * } = \frac{w}{c},\;P^{ * } = \frac{{d^{2} }}{{\lambda c\mu_{0} }}P,\;T^{*} = \frac{{T - T_{2} }}{{T_{1} - T_{2} }}, \hfill \\ \delta = \frac{d}{\lambda },\;C^{*} = \frac{{C - C_{2} }}{{C_{1} - C_{2} }},\;t^{ * } = \frac{c}{\lambda }t,\;h = \frac{H}{d},\;\varepsilon = \frac{b}{d},\;{\text{Re}} = \frac{\rho cd}{{\mu_{0} }},\;\mu^{*} = \frac{\mu }{{\mu_{0} }}. \hfill \\ \end{gathered}$$

In terms of these variables, dropping the star mark for simplicity and considering long wavelength and low-Reynolds number approximation, Eqs. (8–12) become:

18 $$\frac{\partial u}{{\partial r}} + \frac{u}{r} + \frac{\partial w}{{\partial z}} = 0,$$

19 $$\frac{\partial P}{{\partial r}} = 0,$$

20 $$\frac{\partial P}{{\partial z}} = \frac{1}{r}\frac{\partial }{\partial r}\left( {r\,\mu (r,\,z)\frac{\partial w}{{\partial r}}} \right) - \left( {M^{2} + \frac{\mu (r,\,z)}{{Da}}} \right)w,$$

21 $$\left( {\frac{3 + 4R}{{3\Pr }}} \right)\left( {\frac{{\partial^{2} T}}{{\partial r^{2} }} + \frac{1}{r}\frac{\partial T}{{\partial r}}} \right) + Ec\;M^{2} \;w^{2} + Nt\left( {\frac{\partial T}{{\partial r}}} \right)^{2} + Nb\left( {\frac{\partial T}{{\partial r}}\frac{\partial C}{{\partial r}}} \right) = 0,$$

22 $$\frac{{\partial^{2} C}}{{\partial r^{2} }} + \frac{1}{r}\frac{\partial C}{{\partial r}} + \frac{Nt}{{Nb}}\,\left( {\frac{{\partial^{2} T}}{{\partial r^{2} }} + \frac{1}{r}\frac{\partial T}{{\partial r}}} \right) = 0.$$

Thus, the boundary conditions (13) and (14) in their dimensionless form are transformed into:

23 $$w^{\prime } = 0,\;T = C = 1,\;{\text{at}}\;r = \varepsilon .$$

24 $$w = - 1,\;T = C = 0\;{\text{at}}\;r = h = 1 + \varepsilon \sin 2\pi z.$$

The following formula, Eldabe et al.^20^ and Lachiheb^41^, considers the fluid viscosity based on both radial and axial components:

25 $$\mu (r,\,z) = \mathop e\nolimits^{ - \alpha r/h(z)} .$$

This choice is induced by the following physiological phenomena^42^:

1. The chyme viscosity is affected by a excretion of liquids plenty. The latter consists mainly of water and acids and is injected into the intestine lumen from the wall.
2. During the blood transport in the arteries and blood-vessels, the white blood cells and plasma are precipitated in the center while the red blood cells are piled in the boundaries of the wall, resulting in a decrease in the value of the viscosity at the points more closer to the wall.

For $\alpha \, \ll \,$ 1, the formula (19) will tend to the following relation

26 $$\mu (r,\,z) = 1 - \alpha r/h.$$

Morever, the viscosity in the variable case is the viscosity of the base fluid, there are also micro-organism particles present inside the fluid called nanoparticles which are known as the fluid mixture or nanofluid. So, the variable viscosity presented in the current paper of the base fluid depends on the place only, and not the temperature, which means that the thermal physical properties in this study will not change in the presence of both Brownian and the thermophoresis effect. This is because the Brownian motion and the thermophoresis coefficient are not defined by the variable viscosity parameter.

In other side, there are many papers which take the variable viscosity case without considering variable thermophysical properties of the model^21–23,41^.

Equations (14), (15) and (16) are highly non-linear ordinary differential equations. If Da = M = 0 and in the absence of heat and mass transfer, this study tends to the work of Eldabe et al. ^20^.

## Entropy generation analysis

The dimensionless entropy generation can be written as follows^33^:

27 $$Eg = \left( {\frac{\partial T}{{\partial r}}} \right)^{2} + \left( {\frac{3\Pr }{{3 + 4R}}} \right)\,\left( {EcM^{2} w^{2} + Nt\left( {\frac{\partial T}{{\partial r}}} \right)^{2} + Nb\left( {\frac{\partial T}{{\partial r}}\frac{\partial C}{{\partial r}}} \right)} \right),$$

The ratio between heat transfer entropy to total entropy is defined by Bejan number Bn.

## Method of solution

### Exact solution

The exact solutions of Eq. (20) with boundary conditions (23) and (24), can be written as

28 $$\begin{aligned} w(r,z) & = a_{1} r + a_{2} r^{2} + (a_{3} + a_{7} r)\ln r + (a_{4} + a_{6} r)\ln (h - a_{5} r) \\ & \quad + a_{8} \ln (h - (a_{5} - 1)r) + a_{9} Li_{2} \left( {\frac{{a_{5} r}}{h}} \right) + a_{10} , \\ \end{aligned}$$

where $Li_{n} \left( z \right)$ is the polylogarithm function, which is defined by

29 $$Li_{n} \left( z \right) = \frac{{( - 1)^{n - 2} }}{(n - 2)!}\int\limits_{0}^{1} {(\ln (t))^{n - 2} } \ln (1 - zt)dt,$$

### Homotopy perturbation method

The homotopy perturbation technique is a useful method, which can treat many kinds of differential equations systems because it requires only a few steps to obtain semi-analytical solutions for these systems. In addition, it is a combination of the perturbation method and the homotopy analysis method. One of the most important steps in the homotopy perturbation method is to guess an initial solution. Following^43–47^, Eqs. (21) and (22) can be rewritten as follows:

30 $$\begin{aligned} H(p,T) & = L(w) - L(w_{10} ) + pL(w_{10} ) + p\left( {\frac{3\Pr }{{3 + 4R}}} \right)\left( {EcM^{2} w^{2} + Nt\left( {\frac{\partial T}{{\partial r}}} \right)^{2} } \right. \\ & \quad \left. { + Nb\left( {\frac{\partial T}{{\partial r}}\frac{\partial C}{{\partial r}}} \right)} \right), \\ \end{aligned}$$

31 $$H(p,C) = L(C) - L(C_{10} ) + pL(C) + p\left( {\frac{Nt}{{Nb}}\left( {\frac{{\partial^{2} T}}{{\partial r^{2} }} + \frac{1}{r}\frac{\partial T}{{\partial r}}} \right)} \right).$$

With the linear operator. The initial approximations $T_{10}$ and $C_{10}$ can be written as

32 $$T_{10} = C_{10} = \frac{{\ln r - \ln r_{2} }}{{\ln r_{1} - \ln r_{2} }}.$$

Now, we assume that:

33 $$(T,C) = (T_{0} ,C_{0} ) + p(T_{1} ,C_{1} ) + p^{2} (T_{2} ,C_{2} ) + \cdots .$$

Substituting from (33) into (30) and (31), we get the solutions of these equations as:

34 $$\begin{aligned} T(r,z) & = \frac{{\ln \left( {r/r_{2} } \right)}}{{\ln \left( {r_{1} /r_{2} } \right)}} + a_{11} r + a_{12} r^{2} + a_{13} r^{3} + a_{14} r^{4} + (a_{15} + a_{16} r + a_{17} r^{2} + a_{18} r^{3} ) \\ & \quad \times \ln r + a_{19} r^{2} \left( {\ln r} \right)^{2} + (a_{20} + a_{21} r + a_{22} r^{2} + a_{23} r^{3} )\ln (h - a_{5} r) + \\ & \quad + (a_{24} + a_{25} r^{2} )\ln (h - (a_{5} - 1)r) + (a_{26} + a_{27} r^{2} + a_{28} \ln \frac{{a_{5} r}}{h}) \\ & \quad \times \left( {\ln (h - a_{5} r)} \right)^{2} + a_{29} \ln ((a_{5} + 1)r - h) + \left( {a_{30} + a_{31} \ln r} \right)Li_{2} \left( {\frac{{a_{5} r}}{h}} \right) \\ & \quad + a_{32} \ln (h - a_{5} r)Li_{2} \left( {1 - \frac{{a_{5} r}}{h}} \right) + a_{33} Li_{3} \left( {\frac{{a_{5} r}}{h}} \right) + a_{34} Li_{3} \left( {1 - \frac{{a_{5} r}}{h}} \right), \\ \end{aligned}$$

35 $$C(r,z) = \frac{{\ln \left( {r/r_{2} } \right)}}{{\ln \left( {r_{1} /r_{2} } \right)}} + a_{35} + a_{36} \ln r - \frac{Nt}{{Nb}}T_{1} (r,z),$$

### Convergence of homotopy perturbation method

Assume that the solution of Eqs. (21) and (22) can be written as a power series as follows

36 $$n(r,P) = n_{{^{0} }} + Pn_{{^{1} }} + P^{2} n_{{^{2} }} + \cdots .$$

where *n* is one of the physical quantities *T* and *C*. Setting $P = 1$ we obtain the semi-analytical solution of Eqs. (21) and (22) as follows:

37 $$n = \mathop {Lim}\limits_{P \to 1} \left( {n^{0} + Pn^{1} + P^{2} n^{2} + \cdots } \right).$$

The series of Eqs. (21) and (22) are convergent for most of all cases.

The dimensionless volume flow rate, in the fixed frame, is given by

38 $$Q(z,t) = 2\int_{0}^{h} {rw\,dr} ,$$

Now, Nusselt number Nu and Sherwood number Sh are defined, respectively, by

39 $${\text{Nu}} = \left. {\frac{\partial \theta }{{\partial r}}} \right|_{r = h} \;{\text{Sh}} = \left. {\frac{\partial C}{{\partial r}}} \right|_{r = h} .$$

The expressions for Nu and Sh have been calculated by substituting from Eqs. (34) and (35) into Eq. (39) respectively, and they have been evaluated numerically for several values of the parameters of the problem, using the software Mathematica package. The obtained results will be discussed in the next section.

## Results and discussion

In our study, we assumed that the viscosity coefficient varies with both radial coordinate *r* and axial coordinate *z*; moreover, long wavelength and small Reynolds number assumptions restricted our work, while the wave number is neglected. The default values of problem-related parameters are taken as:

$$\begin{gathered} Pz = 0.1, \alpha = 0.3, M = 15, {\text{Da}} = 0.1, R = 1, {\text{Ec}} = 0.2, \Pr = 4.5, Nt = 3.5, \hfill \\ Nb = 1.5, r1 = 0.3, z = 0.8, \varepsilon = 0.1. \hfill \\ \end{gathered}$$

The following values of human small intestine parameters are used^48^

$$d = 1.2 {\text{cm}}, c = 2 {\text{cm}}/{\text{min}}, \;\lambda = 8.1 {\text{cm}}.$$

**Figure 1:**
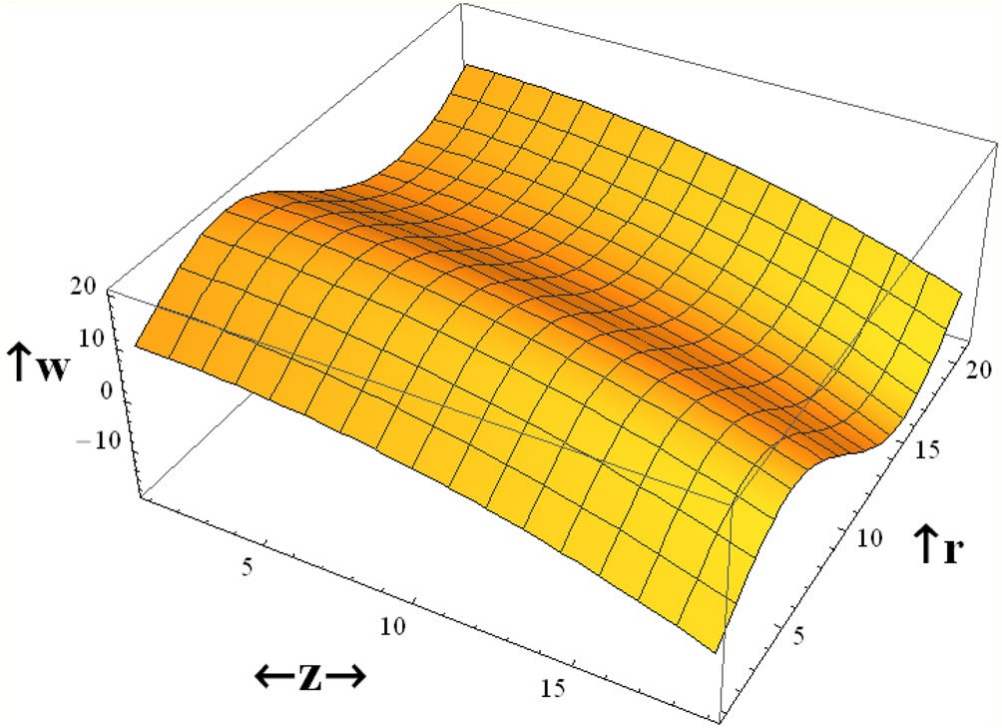
Three-dimensional axial velocity is plotted versus r and z.

**Figure 2:**
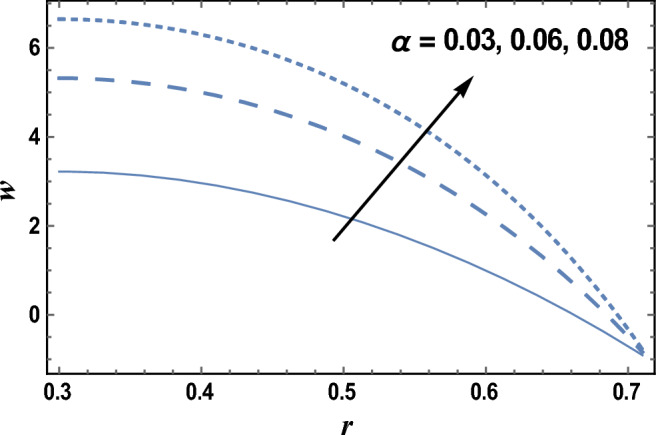
The axial velocity w is plotted with r, for different values of $\alpha$.

**Figure 3:**
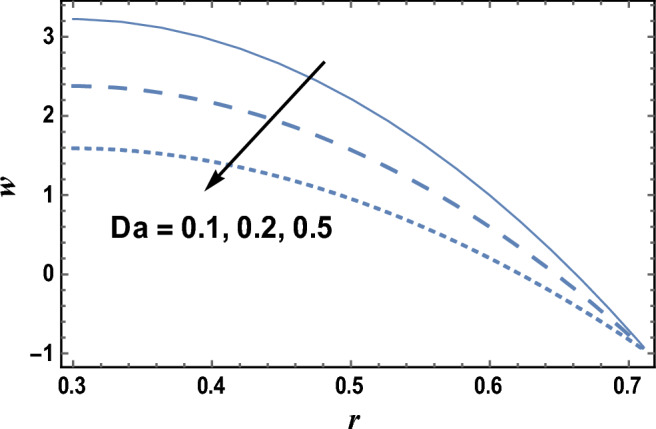
The axial velocity w is plotted with r, for different values of Da.

In Fig. 1, a three-dimensional graph is drawn to illustrate the effects of radial coordinate *r* and axial coordinate *z* on the axial velocity *w*. We observed from this figure that the axial velocity *w* increases with increasing z, while it decreases as r increases. The parameter of viscosity $\user2{ }\alpha$ is affected by the combination of some materials such as crude oil, the temperature, dissolved gas content, and pressure. The viscosity parameter will decrease, when temperature increases, as a result, viscosity measurements are always reported with the temperature at which the measurement is made. The effects of the viscosity parameter $\alpha$ and Darcy number Da on the axial velocity* w* which is a function of the radial coordinate *r* are shown in Figs. 2 and 3, and it is shown that the axial velocity *w* increases by increasing $\alpha$, and the axial velocity increases with *r*, with a relationship that seems like a parabola. While the axial velocity *w* decreases as Da increases as given in Fig. 3. The following clarifies the result in Fig. 2; due to the relation in Eq. (19), it is found that the increment of the viscosity parameter will help the fluid to move easier. Similarly, if we draw the variation of *w* with *r* for different values of the radiation parameter *R*, we will obtain a figure in which the behavior of the curves is the same as that obtained in Fig. 3, except that the obtained curves are very close to those obtained in Fig. 3, but this figure will not be given there to save space.

**Figure 4:**
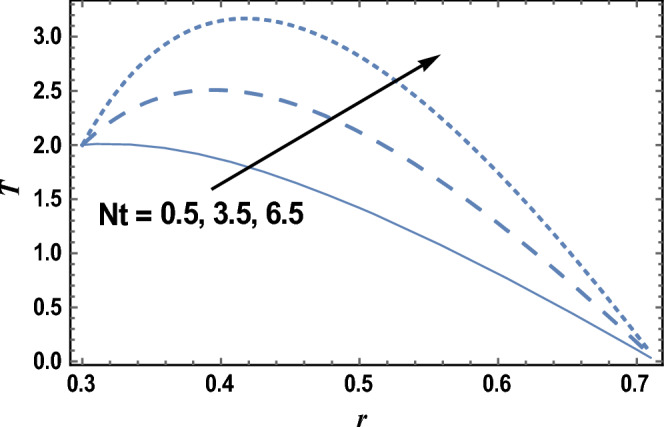
The temperature distribution T is plotted with r, for different values of Nt.

**Figure 5:**
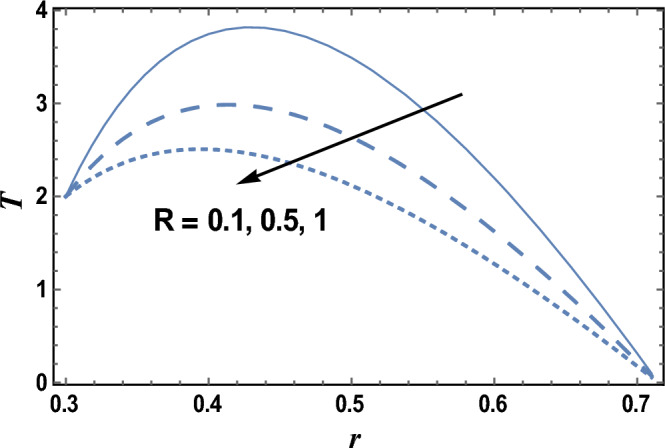
The temperature distribution T is plotted with r, for different values of R.

Thermophoresis or thermo-migration is an original sin that occurs in a blend of transported particles, where the different particle types display different echoes to the temperature gradient force. The effects of the thermophoresis parameter Nt and radiation parameter R on the temperature distribution T which is a function of *r* are shown in Figs. 4 and 5, respectively. It is clear from these figures that the temperature distribution is always negative, and it increases by increasing Nt, while it decreases as R increases. It is also noted that for each value of both Nt and R, there exists a maximum value of T, which its value increases by increasing Nt and decreases by increasing R, and all maximum values occur at. Similar results can be obtained, as in Fig. 4, by drawing *T* versus *r* for various values of Brownian motion parameter Nb, but the figure is not given here to save space. the result in Fig. 5 agrees with the physical expectation and previous definition and agrees with those which are presented by^49^.

**Figure 6:**
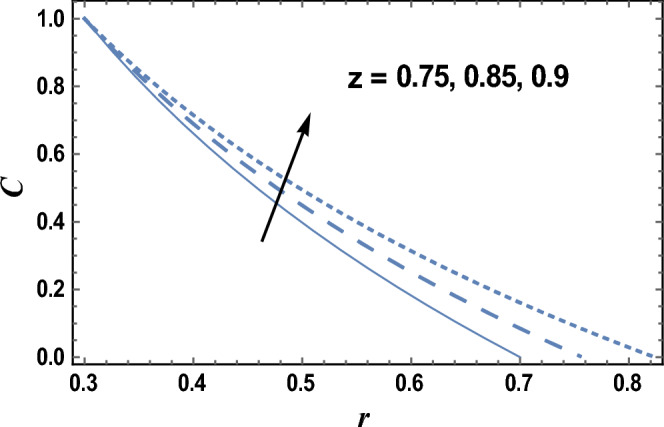
The nanoparticles concentration C is plotted with r, for different values of z.

**Figure 7:**
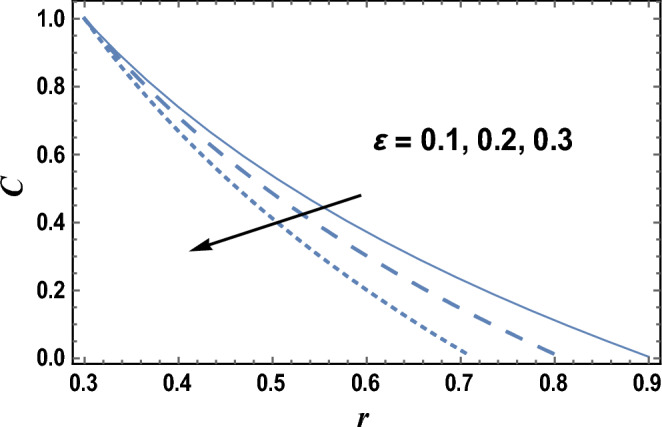
The nanoparticles concentration C is plotted with r, for different values of $\varepsilon$.

Equation (29) evaluates how the nanoparticles concentration distribution C changes with the radial coordinate r. The effects of axial coordinate z and the dimensionless wave amplitude $\varepsilon$ on the nanoparticles concentration distribution C are given in Figs. 6 and 7, respectively. It is found that the nanoparticles concentration increases by increasing z, but it decreases as ${\varvec{\varepsilon}}$ increases. Furthermore, the nanoparticles concentration is always positive and for large values of ${\varvec{\varepsilon}}$ and small values of z, the relation between C and r is a straight line. The effects of other parameters are similar to those obtained in Figs. 6 and 7.

**Figure 8:**
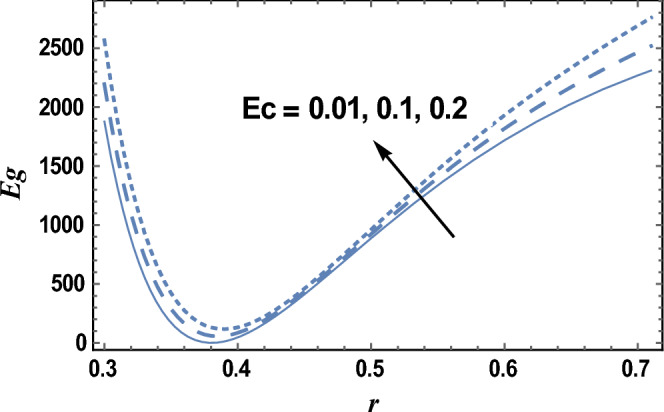
The entropy generation Eg is plotted with r, for different values of Ec.

**Figure 9:**
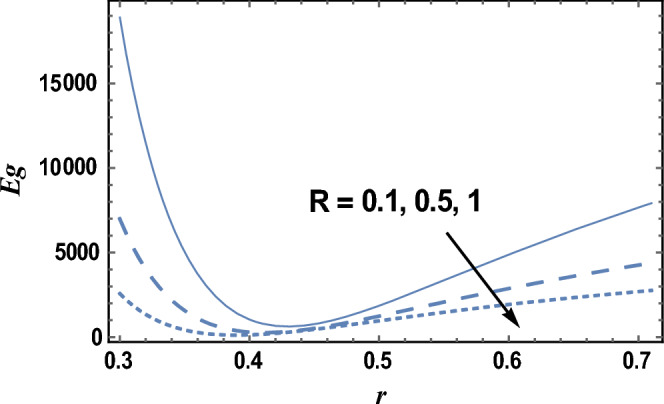
The entropy generation Eg is plotted with r, for different values of R.

**Figure 10:**
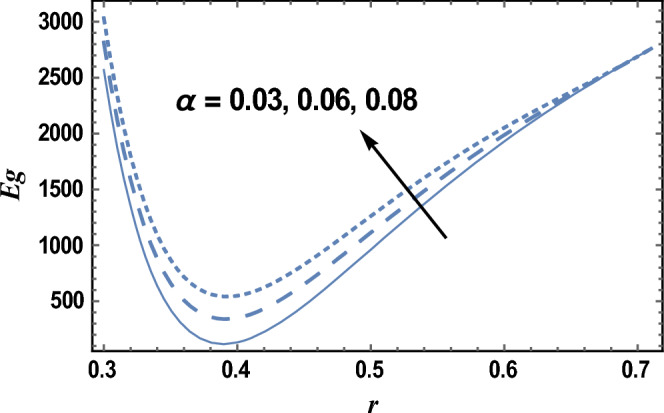
The entropy generation Eg is plotted with r, for different values of $\alpha$.

**Figure 11:**
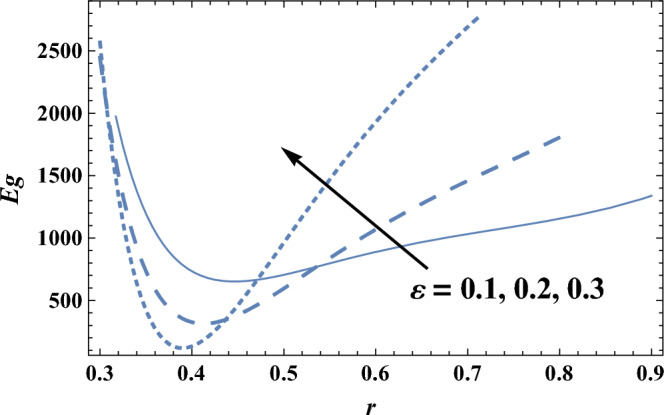
The entropy generation Eg is plotted with r, for different values of $\varepsilon$.

Figure 8 and 9 give the influence of Eckert number Ec, and radiation parameter R on entropy generation Eg, respectively. Therefore, in these figures the Eq. (27) is evaluated by setting z = 0.8 and entropy generation is plotted versus the radial coordinate r. It is noted from these figures that entropy generation increases with the increase of Ec, whereas it decreases as R increases. It is also noted that the entropy generation for different values of Ec and R becomes lower with increasing r and reaches a minimum value (at a finite value of r : r = r_0_) after which it increases. The following clarifies the viscous dissipation effect on entropy generation, namely, the result in Fig. 8. Moreover, all curves for different values of Ec and R intersect at this minimum value. It is well known that the influence of dissipation produces heat due to traction between the particles of fluid, this supplementary heat is a reason for the increase of initial entropy of fluid. This increase in entropy generation causes an additional increment of the force of buoyant. As the buoyant force increases, the fluid velocity increases. So, the bigger traction between the particles of fluid and consequently bigger viscous heating of the fluid. Figure 10 shows the variation of the entropy generation Eg with r for various values of viscosity parameter $\alpha$. It is seen from Fig. 10, that the entropy generation increases with the increase near the outer tube, namely, in the interval r ∈ [0.62, 0.7]; otherwise, it decreases by increasing $\alpha$. Therefore, the behavior of Eg in the interval r ∈ [0.62, 0.7] is opposite to its behavior in the interval r ∈ [0, 0.62]. The effect of the dimensionless wave amplitude $\varepsilon$ on entropy generation is illustrated in Fig. 11. It is found that the effect of $\varepsilon$ on Eg is opposite to the effect of $\alpha$ on Eg given in Fig. 10, with the only difference that, the curves in Fig. 10 are very close to those to each other in the second interval than those obtained in Fig. 11 The other figures are excluded here to save space.

Table 2 presents numerical results for Nusselt number* Nu* and Sherwood number *Sh* for various values of the dimensionless wave amplitude and Brownian motion parameter Nb. It is found from Table 2 that an increase in ${\varvec{\varepsilon}}$ decreases the values of both Nu and Sh. While an increase in Nb gives an opposite behavior to ${\varvec{\varepsilon}}$ in the case of Sh. Moreover, the results in a Table 2 are in agreement with those obtained by ^20^.

**Table 2:** Values of Nu and Sh for various values of $\varepsilon$ and *Nb*.

$\varepsilon$	*Nb*	Nu	Sh
0.0	1.5	− 1.7308	− 0.83058
0.1	1.5	− 2.0814	− 1.00097
0.2	1.5	− 2.5814	− 1.24361
0.3	1.5	− 2.7414	− 1.61190
0.3	2.5	− 2.8434	− 1.58901
0.3	3.5	− 2.8809	− 1.50012
0.3	4.5	− 2.9012	− 1.38897

## Conclusion

In this paper, the influences of both variable viscosity and wave amplitude on MHD peristaltic flow of Newtonian fluid between two co-axial cylinders under the consideration of long wavelength and the law-Reynolds number have been studied. In our analysis, we are taking into account the effects of both porous medium, Ohmic dissipation, and radiation. The analytical expressions are constructed for the velocity, temperature and nanoparticles concentration distributions. The present analysis can avail as a model which may help in understanding the mechanics of physiological flows^50–52^. The effects of various pertinent parameters on the flow are discussed through numerical computations. The main findings can be summarized as follows:

1. The axial velocity *w* decreases with the increase in each of $\varepsilon$, R and *Da*, whereas it increases as $\alpha$, Pr and *M* increase.
2. All curves of the axial velocity *w* for different values of $\alpha$, R, *Da*, $\varepsilon$, Pr and *M* don’t intersect at the boundary of the inner tube, then decrease with increasing r and they intersect at the boundary of the outer tube.
3. The temperature increases with the increase each of Pr, *Nt*, Nb and *Ec* whereas it decreases as both R and $\varepsilon$ increase.
4. By increasing the radial coordinate r, the temperature T for different values of problem physical parameters becomes greater and ends up at a maximum value in the middle of the tubes.
5. The nanoparticles concentration C has an opposite behavior compared to the temperature behavior.

## Data Availability

The datasets generated and/or analyzed during the current study are not publicly available due to [All the required data are only with the corresponding author] but are available from the corresponding author on reasonable request.

## List of symbols

*a*: The radius of inner tube (L)
*B*: The magnetic field $= (B_{0} ,0,0)$
*b*: The dimensional wave amplitude
c: The propagation velocity along ${\text{z}}$ direction
*c*_*p*_: The specific heat at constant pressure
*C*: The fluid nanoparticles concentration (M)
*C*_1_: Nanoparticles concentration at $r = r_{1}$ (M)
*C*_2_: Nanoparticles concentration at $r = r_{2}$ (M)
*d*: The radius of outer tube (L)
Da: Darcy number $= \frac{K}{{d^{2} }}$
*D*_B_: Brownian diffusion coefficient
*D*_T_: Thermophoretic diffusion coefficient
Ec: Eckert number $= \frac{{c^{2} }}{{c_{P} (T_{1} - T_{2} )}}$
*k*: Coefficient of thermal conductivity
*K*: The permeability parameter
*M*: Magnetic field parameter $= \frac{{\sigma B_{0}^{2} d^{2} }}{{\mu_{0} }}$
*N*_*b*_: Brownian motion parameter $\, = \frac{{D_{B} \,(C_{1} - C_{2} )\,\left( {\rho c} \right)_{p} }}{{\left( {\rho c} \right)_{f} \,c\,d}}$
*N*_*t*_: The thermophoresis parameter $= \frac{{D_{T} \,(T_{1} - T_{2} )\,\left( {\rho c} \right)_{p} }}{{T_{1} \,\left( {\rho c} \right)_{f} \,c\,d}}$
*P*: The fluid pressure
Pr: Prandtl number $= \frac{{\mu_{0} \,c_{P} }}{k}$
$q_{r}$: The radiative heat flux
*r*: Radial coordinate (L)
*R*: Radiation parameter $$= \frac{{4\sigma^{ * } T_{w}^{3} }}{{k\,k_{R} }}$$
Re: Reynolds number $= \frac{\rho \,c\,d}{{\mu_{0} }}$
*t*: The time (t)
*T*: The fluid temperature
*T*_1_: Temperature at $$r = r_{1}$$
*T*_2_: Temperature at $$r = r_{2}$$
*u*: Radial velocity (L t^−1^)
*w*: Axial velocity (L t^−1^)
*z*: Axial coordinate (L)

## Greek symbols

$\alpha$: The viscosity parameter
*σ*: The electrical conductivity
*ε*: The dimensionless wave amplitude
λ: The wavelength
$\mu$: The viscosity of the fluid
$\mu_{0}$: The fluid viscosity at $r = r_{1}$
$\rho$: The fluid density
$(\rho c)_{p}$: Effective heat capacity of the nanoparticle material

## References

[1] Ismael AM, Eldabe NTM, Abou-zeid MY, Elshabouri SM (2022). Thermal micropolar and couple stresses effects on peristaltic flow of biviscosity nanofluid through a porous medium. Sci. Rep..

[2] Ouaf ME, Abou-zeid MY (2021). Hall currents effect on squeezing flow of non-Newtonian nanofluid through a porous medium between two parallel plates. Case Stud. Therm. Eng..

[3] Eldabe NTM, Abou-zeid MY, Mohamed MAA, Abd-Elmoneim MM (2021). MHD peristaltic flow of non-Newtonian power-law nanofluid through a non-Darcy porous medium inside a non-uniform inclined channel. Arch. Appl. Mech..

[4] Abou-zeid MY (2011). Magnetohydrodynamic boundary layer heat transfer to a stretching sheet including viscous dissipation and internal heat generation in a porous medium. J. Porous Med..

[5] Riaz A, Ellahi R, Nadeem S (2014). Peristaltic transport of a Carreau fluid in a compliant rectangular duct. Alex. Eng. J..

[6] Akram S, Athar M, Saeed K (2021). Hybrid impact of thermal and concentration convection on peristaltic pumping of Prandtl nanofluids in non-uniform inclined channel and magnetic field. Case Stud. Therm. Eng..

[7] Abou-zeid MY (2015). Homotopy perturbation method to gliding motion of bacteria on a layer of power-law nanoslime with heat transfer. J. Comput. J. Comput. Theor. Nanosci..

[8] Khan MI, Waqas M, Hayat T, Khan MI, Alsaedi A (2017). Chemically reactive flow of upper-convected Maxwell fluid with Cattaneo–Christov heat flux model. J. Braz. Soc. Mech. Sci. Eng..

[9] Eldabe NT, Abou-zeid MY (2018). Radially varying magnetic field effect on peristaltic motion with heat and mass transfer of a non-Newtonian fluid between two co-axial tubes. Therm. Sci..

[10] Eldabe NTM, Abou-zeid MY, Elshabouri SM, Salama TN, Ismael AM (2022). Ohmic and viscous dissipation effects on micropolar non-Newtonian nanofluid Al2O3 flow through a non-Darcy porous media. Int. J. Appl. Electromagn..

[11] Sami AR, Khan U, Farid S, Khan MI, Sun TC, Abbasi A, Khan MI, Malik MY (2021). Thermal activity of conventional Casson nanoparticles with ramped temperature due to an infinite vertical plate via fractional derivative approach. Case Stud. Therm. Eng..

[12] Mohamed MA, Abou-zeid MY (2019). MHD peristaltic flow of micropolar Casson nanofluid through a porous medium between two co-axial tubes. J. Porous Med..

[13] Eldabe NTM, Moatimid GM, Abou-zeid M, Elshekhipy AA, Abdallah NF (2020). Instantaneous thermal-diffusion and diffusion-thermo effects on Carreau nanofluid flow over a stretching porous sheet. J. Adv. Res. Fluid Mech. Therm. Sci..

[14] Eldabe NTM, Abou-zeid MY, Abosaliem A, Alana A, Hegazy N (2021). Homotopy perturbation approach for Ohmic dissipation and mixed convection effects on non-Newtonian nanofluid flow between two co-axial tubes with peristalsis. Int. J. Appl. Electromag. Mech..

[15] Khan MI, Hayat T, Afzal S, Khan MI, Alsaedi A (2020). Theoretical and numerical investigation of Carreau-Yasuda fluid flow subject to Soret and Dufour effects. Comput. Methods Programs Biomed..

[16] Mansour HM, Abou-zeid MY (2019). Heat and mass transfer effect on non-Newtonian fluid flow in a non-uniform vertical tube with peristalsis. J. Adv. Res. Fluid Mech. Therm. Sci..

[17] Eldabe NT, Shaaban AA, Abou-zeid MY, Ali HA (2015). Magnetohydrodynamic non-Newtonian nanofluid flow over a stretching sheet through a non-Darcy porous medium with radiation and chemical reaction. J. Comput. Theor. Nanosci..

[18] El Ouaf M, Abou-zeid M (2021). Electromagnetic and non-Darcian effects on a micropolar non-Newtonian fluid boundary-layer flow with heat and mass transfer. Int. J. Appl. Electromagn. Mech..

[19] Eldabe NT, Abou-zeid MY, El-Kalaawy OH, Moawad SM, Ahmed OS (2021). Electromagnetic steady motion of Casson fluid with heat and mass transfer through porous medium past a shrinking surface. Therm. Sci..

[20] Eldabe NT, Abo-Seida OM, Abd El Naby AE, Ibrahim M (2022). Effects of bivariation viscosity and magnetic field on trapping in a uniform tube with peristalsis. Inf. Sci. Lett..

[21] Nadeem S, Hayat T, Akbar NS, Malik MY (2009). On the influence of heat transfer with variable viscosity. Int. J. Heat Mass Transf..

[22] Asghar S, Hussain Q, Hayat T (2013). Peristaltic flow of reactive viscous fluid with temperature dependent viscosity. Math. Comput. Appl..

[23] Eldabe NT, Moatimid GM, Abouzeid MY, ElShekhipy AA, Abdallah NF (2020). A semianalytical technique for MHD peristalsis of pseudoplastic nanofluid with temperature- dependent viscosity: Application in drug delivery system. Heat Transf.-Asian Res..

[24] Choi SUS, Siginer DA, Wang HP (1995). Enhancing thermal conductivity of fluids with nanoparticles, developments and applications of non-Newtonian flows. ASME FED.

[25] Shafiq A, Rasool G, Khalique CM, Aslam S (2020). Second grade bioconvective nanofluid flow with buoyancy effect and chemical reaction. Symmetry.

[26] Eldabe NTM, Rizkallah RR, Abou-zeid MY, Ayad VM (2020). Thermal diffusion and diffusion thermo effects of Eyring- Powell nanofluid flow with gyrotactic microorganisms through the boundary layer. Heat Transf.: Asian Res..

[27] Eldabe NTM, Rizkallah RR, Abou-zeid MY, Ayad VM (2022). Effect of induced magnetic field on non-Newtonian nanofluid Al2IO3 motion through boundary-layer with gyrotactic microorganisms. Therm. Sci..

[28] Eldabe NTM, Moatimid GM, Abou-zeid M, Elshekhipy AA, Abdallah NF (2021). Semi-analytical treatment of Hall current effect on peristaltic flow of Jeffery nanofluid. Int. J. Appl. Electromag. Mech..

[29] Abou-zeid MY, Shaaban AA, Alnour MY (2015). Numerical treatment and global error estimation of natural convective effects on gliding motion of bacteria on a power-law nanoslime through a non-Darcy porous medium. J. Porous Med..

[30] Abou-zeid MY, Mohamed MAA (2017). Homotopy perturbation method for creeping flow of non-Newtonian power-law nanofluid in a nonuniform inclined channel with peristalsis. Zeitschrift für Naturforsch A.

[31] Song Y, Hamid A, Khan MI, Gowd RJP, Kumard RN, Prasannakumara BC, Khan SU, Khan MI, Malik MY (2021). Solar energy aspects of gyrotactic mixed bioconvection flow of nanofluid past a vertical thin moving needle influenced by variable Prandtl number. Chaos Sol. Fract..

[32] Abou-zeid M (2016). Effects of thermal-diffusion and viscous dissipation on peristaltic flow of micropolar non-Newtonian nanofluid: Application of homotopy perturbation method. Res. Phys..

[33] Ouaf ME, Abou-Zeid MY, Younis YM (2022). Entropy generation and chemical reaction effects on MHD non-Newtonian nanofluid flow in a sinusoidal channel. Int. J. Appl. Electromagn. Mech..

[34] Khan MI, Hayat T, Qayyum S, Khan MI, Alsaedi A (2018). Entropy generation (irreversibility) associated with flow and heat transport mechanism in Sisko nanomaterial. Phys. Lett. A.

[35] Eldabe NT, Abo-zeid MY, Younis YM (2017). Magnetohydrodynamic peristaltic flow of Jeffry nanofluid with heat transfer through a porous medium in a vertical tube. Appl. Math. Inf. Sci..

[36] Abou-zeid MY (2017). Homotopy perturbation method for MHD non-Newtonian nanofluid flow through a porous medium in eccentric annuli in peristalsis. Therm. Sci..

[37] Eldabe NT, Elshabouri S, Elarabawy H, Abouzeid MY, Abuiyada AJ (2022). Wall properties and Joule heating effects on MHD peristaltic transport of Bingham non-Newtonian nanofluid. Int. J. of Appl. Electromagn. Mech..

[38] Abou-zeid MY (2019). Implicit homotopy perturbation method for MHD non-Newtonian nanofluid flow with Cattaneo–Christov heat flux due to parallel rotating disks. J. Nanofluids.

[39] Abuiyada AJ, Eldabe NT, Abouzeid MY, Elshabouri S (2022). Effects of thermal diffusion and diffusion thermo on a chemically reacting MHD peristaltic transport of Bingham plastic nanofluid. J. Adv. Res. Fluid Mech. Therm. Sci..

[40] Mohamed YM, El-Dabe NT, Abou-zeid MY, Oauf ME, Mostapha DR (2022). Effects of thermal diffusion and diffusion thermo on a chemically reacting MHD peristaltic transport of Bingham plastic nanofluid. J. Adv. Res. Fluid Mech. Therm. Sci..

[41] Lachiheb M (2014). Effect of coupled radial and axial variability of viscosity on the peristaltic transport of Newtonian fluid. Appl. Math. Comput..

[42] Lachiheb M (2018). A bivariate viscosity function on the peristaltic motion in an asymmetric channel. ARPN J. Eng. Appl Sci..

[43] Farooq U, Hayat T, Zhao YL, Alsaedi A, Liao S (2015). Application of the HAM-based mathematica package BVPh 2.0 on MHD Falkner–Skan flow of nano-fluid. Comput. Fluids.

[44] Farooq U, Xu H (2014). Free convection nanofluid flow in the stagnation- point region of a three-dimensional body. Sci. World J..

[45] Farooq U, Hayat T, Alsaedi A, Liao S (2014). Heat and mass transfer of two-layer flows of third-grade nano-fluids in a vertical channel. Appl. Math. Comput..

[46] Farooq U, Zhi-Liang L (2014). Nonlinear Heat transfer in a two-layer flow with nanofluids by OHAM. ASME J. Heat Transf..

[47] Lu D, Farooq U, Hayat T, Rashidi MM, Ramzan M (2018). Computational analysis of three layer fluid model including a nanomaterial layer. Int. J. Heat Mass Transf..

[48] Eldabe NT, Abou-zeid M (2014). Magnetohydrodynamic peristaltic flow with heat and mass transfer of micropolar biviscosity fluid through a porous medium between two co-axial tubes. Arab. J. Sci. Eng..

[49] El-Dabe NT, Abouzeid MY, Ahmed OS (2020). Motion of a thin film of a fourth grade nanofluid with heat transfer down a vertical cylinder: Homotopy perturbation method application. J. Adv. Res. Fluid Mech. Therm. Sci..

[50] Eldabe NTM, Abouzeid MY, Ali HA (2020). Effect of heat and mass transfer on Casson fluid flow between two co-axial tubes with peristalsis. J. Adv. Res. Fluid Mech. Therm. Sci..

[51] Eldabe NTM, Hassan MA, Abouzeid MY (2015). Wall properties effect on the peristaltic motion of a coupled stress fluid with heat and mass transfer through a porous medi. J. Eng. Mech..

[52] Eldabe NTM, Abouzeid MY, Shawky HA (2021). MHD peristaltic transport of Bingham blood fluid with heat and mass transfer through a non-uniform channel. J. Adv. Res. Fluid Mech. Therm. Sci..

